# Culprit lesion characteristics and prognosis in STEMI with cold onset: an OCT study

**DOI:** 10.1038/s44325-024-00019-5

**Published:** 2024-10-01

**Authors:** Qianhui Sun, Xing Luo, Boling Yi, Chen Zhao, Minghao Liu, Ming Zeng, Haibo Jia, Bo Yu

**Affiliations:** 1 Harbin Medical University, 150081 Harbin, PR China; 2 Department of Cardiology, The 2nd Affiliated Hospital of Harbin Medical University, 150001 Harbin, PR China; 3 National Key Laboratory of Frigid Zone Cardiovascular Diseases, 150001 Harbin, PR China

**Keywords:** Cardiology, Cardiovascular diseases

## Abstract

Cold temperature exposure is associated with increased cardiovascular morbidity. However, limited research has explored plaque characteristics and prognosis in ST-segment elevation myocardial infarction (STEMI) patients diagnosed in cold temperatures. In the current study, 517 STEMI patients who underwent coronary optical coherence tomography examination were included and divided according to a median of the ambient temperature(11.5 °C). Our result shows that the cold temperature group exhibited higher proportions of plaque rupture, 78.1%, compared to 68.8% in the warm temperatures group. Besides, patients in the cold temperature group showed thinner minimum fibrous cap thickness (60.0 vs. 70.0 μm, *p* = 0.035). Furthermore, the cold temperature group showed a higher incidence rate of major adverse cardiac events (MACE), which includes cardiac death, recurrent nonfatal myocardial infarction, stroke, or hospitalization for heart failure (15.7% vs. 9.7%, *p* = 0.041). Moreover, cold temperature exposure at the onset independently predicted MACE (HR1.83 [95%CI 1.06–3.14], *p* = 0.029).

## Introduction

Numerous studies have demonstrated that exposure to extreme temperatures is associated with increased mortality in large populations, especially cold exposure^1–4^. Currently, cardiovascular-related diseases, including ischemic heart disease, which are some of the leading causes of death in the world, are also closely related to extreme weather^1,5,6^. However, although both cold exposure and heat exposure increase the risk of cardiovascular death, the effect of cold exposure is stronger^1^. Several studies found that only cold exposure is associated with an increased prevalence of cardiovascular diseases^7^.

Current research has highlighted that cold exposure has effects on the coagulation system, inflammatory response, oxidative stress, and other aspects^8–10^. Individuals with coronary heart disease at cold temperatures also exhibited higher syntax scores and more severe atherosclerosis^11^. Moreover, previous studies have explored the differences in coronary culprit plaque characteristics associated with different onset seasons^12–14^. However, these studies often involved populations exposed to higher ambient temperatures, potentially limiting the generalizability of the results to colder environments. ST-segment elevation myocardial infarction (STEMI) is the most high-risk form of acute coronary syndrome (ACS) and is associated with the poorest prognosis^15^. There is still a lack of data to explore the impact of ambient temperature on morphologic features in the STEMI population in cities that typically experience long, cold temperatures for the whole year.

Therefore, the purpose of this study is as follows: (1) To explore the differences in plaque characteristics observed by optical coherence tomography (OCT) in STEMI patients who develop symptoms in cold and warm ambient temperatures. (2) To compare the differences in prognosis within 3 years between patients with STEMI who develop cold and warm temperature environments.

## Results

### Baseline clinical characteristics and angiographical findings

The median temperature of all patients on the day of illness onset was 11.5 °C. The highest temperature is 31 °C, and the lowest temperature is −29.5 °C. Figure S1 shows the number of patients at different ambient temperatures. Among them, 270 patients were divided into the cold temperature group, whose temperature was lower than the ambient median temperature on the day of onset, and 247 patients were in the warm temperature group. Baseline demographic characteristics, risk factors, and angiographical characteristics are shown in Table 1. Compared with patients in the warm temperature group, patients in the cold temperature group showed higher hs-CRP (5.4 vs. 4.1 mg/dl, *p* = 0.014). No statistical differences were found in the blood pressure on admission and other laboratory indicators in both groups. The comparability of stent implantation strategy, balloon angioplasty utilization, and drug treatment strategies was observed. In contrast to the warm temperature group, a higher percentage of patients in the cold temperature group exhibited TIMI blood flow of 0 or 1(160(59.0%) vs.122(49.8%), *p* = 0.042).

**Table 1:** Baseline clinical and angiographic characteristics of the study cohort

Variables	Cold temperature group (*n* = 270)	Warm temperature group (*n* = 247)	*p*
Male, *n* (%)	189 (70.0)	180 (72.9)	0.496
Age, years	58.2 ± 11.7	57.8 ± 11.9	0.709
Hypertension, *n* (%)	117 (43.3)	108 (43.7)	0.929
Heart rate	78.0 (67.0–86.0)	79.5 (67.0–99.0)	0.512
Systolic pressure, mmHg	130 (114–146)	130 (114–145)	0.982
Diastolic pressure, mmHg	80 (70–90)	80 (72–90)	0.423
Dyslipidemia, *n* (%)	172 (63.7)	157 (63.6)	0.973
Diabetes mellitus, *n* (%)	87 (32.2)	92 (37.2)	0.230
Current smoker, *n* (%)	142 (52.6)	146 (59.1)	0.156
Pre-PCI, *n* (%)	4 (1.5)	7 (2.8)	0.366
LVEF, %	61 (54–62)	61 (54–63)	0.773
Hemoglobin, g/dl	145.0 (134.0–156.0)	146.0 (134.0–158.0)	0.961
Platelet count, 10^3^/μl	239.0 (193.5–283.3)	231.5 (195.0–271.8)	0.338
White blood count, 10^3^/μl	11.2 (8.9–13.8)	11.4 (9.5–14.1)	0.339
hs-CRP, mg/dl	5.4 (2.3–10.7)	4.1 (1.8–9.2)	0.014
HbA1c, %	5.8 (5.5–6.4)	5.8 (5.5–6.6)	0.611
Total cholesterol, mg/dl	182.4 (155.1–211.9)	178.7 (152.7–205.7)	0.933
Triglyceride, mg/dl	121.3 (85.0–174.4)	126.6 (87.7–193.0)	0.510
LDL-C, mg/dl	111.4 (89.7–135.3)	107.1 (88.9–132.3)	0.882
HDL-C, mg/dl	52.2 (44.1–63.4)	49.5 (43.7–59.2)	0.164
NT-proBNP, pg/ml	145.5.0 (48.0–529.5)	119.0 (45.0–449.0)	0.386
Peak Troponin I, ng/ml	59.7 (17.4–125.8)	53.8 (15.1–109.2)	0.641
BMI, kg/m^2^	24.9 ± 3.8	24.8 ± 3.6	0.825
PCI with drug-eluting stents, *n* (%)	140 (51.9)	114 (46.2)	0.218
*Medications at discharge*			
Aspirin	262 (97.0)	243 (98.4)	0.388
Clopidogrel	91 (33.7)	70 (28.3)	0.216
Ticagrelor	175 (64.8)	173 (70.0)	0.233
Statins	262 (97.0)	240 (97.2)	1.000
β-receptor blocker	167 (61.9)	163 (66.0)	0.360
ACEI/ARB	134 (49.6)	116 (47.0)	0.597
*Culprit vessel, n (%)*			0.242
LAD	138 (51.1)	129 (52.2)	
RCA	88 (32.6)	90 (36.4)	
LCX	44 (16.3)	28 (11.3)	
*Killip, n (%)*			0.129
I	265 (98.0)	245 (99.2)	
II	4 (1.5)	0 (0)	
III	1 (0.4)	2 (0.8)	
TIMI ≦ 1, *n* (%)	160 (59.0)	122 (49.8)	0.042
balloon angioplasty, *n* (%)	124 (45.9)	96 (38.9)	0.105
thrombectomy, *n* (%)	215 (79.6)	182 (74.6)	0.206
MLD, mm	1.1 (0.8–1.4)	1.1 (0.8–1.5)	0.151
RLD, mm	2.7 (2.4–3.1)	2.8 (2.4–3.2)	0.268
DS, %	60 (50.0–70.0)	59.5 (46–68)	0.134
Lesion length, mm	14.2 (11.4–18.4)	14.9 (11.8–18.6)	0.416
Final MLD, mm	1.8 (1.2–2.6)	2.0 (1.3–2.5)	0.263
*Final TIMI, n (%)*			0.876
TIMI = 2	23 (8.5)	22 (8.9)	
TIMI = 3	247 (91.5)	225 (91.1)	
TMPFC > 95.5, *n* (%)	64 (23.7)	49 (19.8)	0.288

Values expressed as *n* (%), mean ± SD, or median (25th–75th percentiles). A *p*-value < 0.05 was considered statistically significant.

*HDL-C* high-density lipoprotein cholesterol, *hs-CRP* high-sensitive C-reactive protein, *DS* diameter stenosis, *LAD* left anterior descending artery, *LCX* left circumflex artery, *LDL-C* low-density lipoprotein cholesterol, *LVEF* left ventricular ejection fraction, *MLD* minimal lumen diameter, *NT-proBNP* N-terminal-pro-brain natriuretic peptide, *PCI* percutaneous coronary intervention, *RCA* right coronary artery, *RLD* reference lumen diameter, *TIMI* thrombolysis in myocardial infarction, *TMPFC* thrombolysis in myocardial infarction myocardial perfusion frame count.

### OCT findings

Baseline OCT characteristics are shown in Table 2. Patients in the cold temperature group showed a higher incidence of plaque rupture (78.1%), while plaque erosion was 21.1%. The prevalence of calcified nodules was found to be 0.7%. On the other hand, in the warm temperature group, 68.8% of patients experienced plaque rupture, while 30.4% showed plaque erosion. The occurrence of calcified nodules was 0.8% (*p* = 0.036). Moreover, the minimum lumen area was smaller (1.6 vs. 1.7 mm^2^, *p* = 0.044), and the minimum fibrous cap thickness of the culprit plaque was significantly thinner (60.0 vs. 70.0 μm, *p* = 0.035) in the cold temperature group (Fig. S2). Besides, although no statistical difference was reached, the culprit plaques were more likely to be lipid plaques (87.0% vs. 81.4%, *p* = 0.077). Another interesting finding is that, as shown in Fig. S3, as the ambient temperature decreases, the minimum fibrous cap thickness of patients also decreases (*r* = 0.10, *p* = 0.045).

**Table 2:** OCT characteristics of culprit plaque between cold temperature group and warm temperature group

Variables	Cold temperature group (*n* = 270)	Warm temperature group (*n* = 247)	*p*
*Plaque type*			0.036
Plaque rupture, *n* (%)	211 (78.1)	170 (68.8)	
Plaque erosion, *n* (%)	57 (21.1)	75 (30.4)	
Calcified nodule, *n* (%)	2 (0.7)	2 (0.8)	
Lipid plaque, *n* (%)	235 (87.0)	201 (81.4)	0.077
Macrophage, *n* (%)	252 (93.3)	225 (91.1)	0.341
Calcification, *n* (%)	154 (57.0)	123 (49.8)	0.099
Microvessel, *n* (%)	61 (22.6)	51 (20.6)	0.592
Cholesterol crystal, *n* (%)	69 (25.6)	50 (20.2)	0.152
MLA, mm^2^	1.6 (1.3–2.2)	1.7 (1.3–2.5)	0.044
AS, %	74.0 (64.0–81.7)	73.0 (62.5–79.4)	0.214
Thinnest FCT, μm	60.0 (50.0–80.0)	70.0 (50.0–90.0)	0.035
Mean lipid arc, deg	176.9 (150.0–207.6)	173.3 (148.8–211.1)	0.837
Maximal lipid arc, deg	290.3 (229.1–360.0)	283.6 (214.9–360.0)	0.483
Lipid length, mm	11.2 (8.3–15.2)	10.2 (7.0–14.3)	0.107

Values expressed as *n* (%), mean ± SD, or median (25th–75th percentiles). A *p*-value < 0.05 was considered statistically significant.

*AS* area stenosis, *FCT* fibrous cap thickness, *MLA* minimal lumen area, *MLD* minimal lumen diameter.

To further explore the influence of lower ambient temperature on plaque characteristics, we further set the 10th percentile of the daily minimum temperature as the extremely cold temperature group (less than −17.5 °C). We compared the ambient environment of the included population according to extreme cold temperatures and non-extreme cold temperatures (Table S1). The results show similar findings to previous studies: patients in the extreme cold temperature group had a higher proportion of plaque rupture and a thinner fibrous cap thickness.

### The prognostic implications of disease onset ambient temperature

**Fig. 1:**
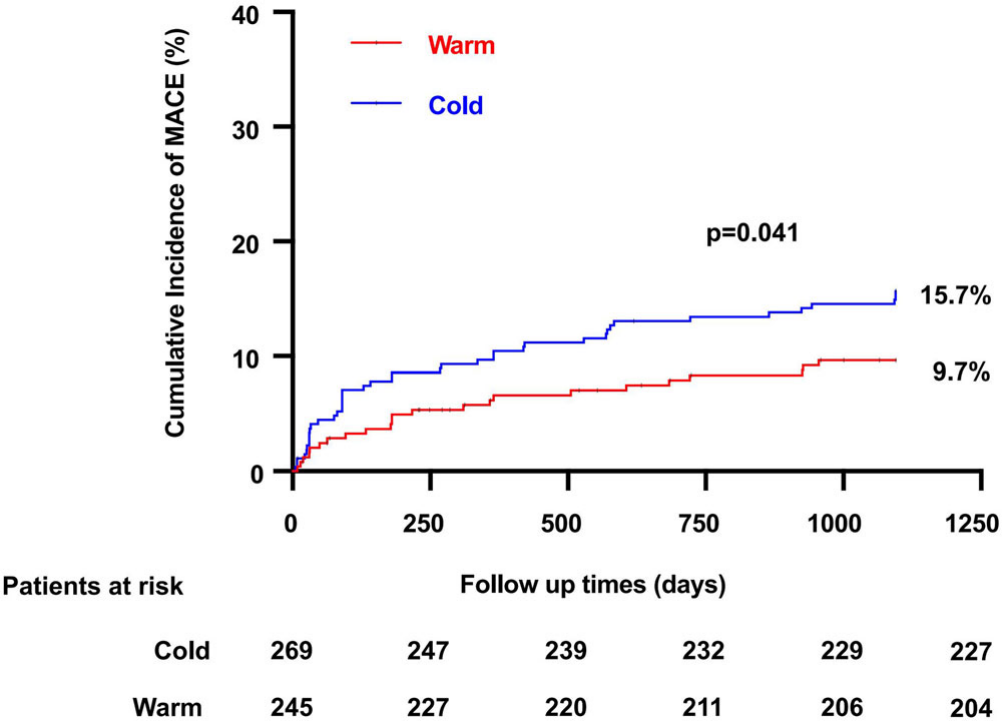
The Kaplan–Meier curve for MACE comparing warm temperature group vs cold temperature group. Kaplan–Meier curves for unadjusted incidence of MACE during the 3-year follow-up period showing the accumulated incidence for MACE. MACE major adverse cardiac events.

A total of three patients died during hospitalization, two of whom were in the cold temperature group and one in the warm temperature group. Major adverse cardiac events (MACE) are a composite of cardiac death, recurrent nonfatal myocardial infarction (MI) or stroke, or hospitalization for heart failure (HF). A comparison of Kaplan–Meier MACE event estimates for the remaining two groups of patients during 3 years of follow-up is shown in Table S2, Figs. 1, and S4. Patients in the cold temperature group experienced 42 MACE events (15.7%), 14 cardiac deaths (5.2%), 11 nonfatal MI or stroke (4.2%), and 21 rehospitalizations for HF (8.0%). Patients in the warm temperature group experienced 23 MACE events (9.7%), 10 cardiac deaths (4.2%), 6 nonfatal myocardial infarctions or strokes (2.6%), and 9 rehospitalizations for HF (3.8%).

Besides, patients have been found to experience worse prognostic clinical outcomes and higher rates of MACE events in the cold temperature group compared to those in the warm temperature group (15.7% vs. 9.7%; *p* = 0.041). Subsequent multivariable Cox proportional hazards analysis revealed that exposure to cold temperature at the onset remained a significant independent predictor of 3-year MACE events (hazard ratio 1.83 [95% confidence interval 1.06–3.14], *p* = 0.029) and HF rehospitalization (hazard ratio 2.44 [95% confidence interval 1.01–5.85], *p* = 0.047) (Table 3).

**Table 3:** Cox proportional hazard analysis comparing cold temperature group and warm temperature group

Event	Unadjusted model		Adjusted model	
	HR (95%CI)	*p*	HR (95%CI)	*p*
MACE	1.69 (1.02–2.81)	0.043	1.83 (1.06–3.14)	0.029
Cardiac death	1.40 (0.59–3.33)	0.443	1.24 (0.55–2.80)	0.600
Nonfatal MI/Stroke	1.64 (0.61–4.43)	0.332	1.76 (0.64–4.85)	0.273
Rehospitalizations for HF	2.15 (0.98–4.69)	0.055	2.44 (1.01–5.85)	0.047

Age, sex, diabetes mellitus, dyslipidemia, hypertension, N-terminal pro-brain natriuretic peptide (NT-proBNP), high-sensitivity C-reactive protein (hs-CRP), and percutaneous coronary intervention (PCI) with drug-eluting stents were adjusted.

*MACE* major adverse cardiac events, *MI* myocardial infarction, *HF* heart failure.

## Discussion

This study attempts to explore the differences in culprit plaque lesion morphology based on ambient temperature among STEMI patients in city which usually experience longer cold temperatures whole year and explore the impact of low ambient temperature on patients’ outcomes. The main findings of this study are as follows: (1) Patients in the cold temperature group exhibit higher hs-CRP, higher proportion of plaque rupture, smaller minimum lumen area, and thinner minimum fibrous cap thickness; (2) STEMI patients diagnosed in cold environments have a higher incidence of 3-year MACE events and rehospitalization due to HF. Therefore, these findings suggest that STEMI patients who develop symptoms in cold weather have unique coronary artery disease characteristics and are at higher risk of recurrence of adverse events.

hs-CRP is an inflammatory predictor that is associated with platelet hyperreactivity^16^, macrophage M1 polarization, and the promotion of the infiltration of macrophages into blood vessels^17^. These pathological processes are all closely related to the process of coronary atherosclerosis formation and progress. Moreover, coronary atherosclerosis is characterized as a chronic local inflammation of the coronary arteries. Therefore, elevated levels of inflammation may worsen the severity of the plaque^18^. Previous studies exploring the association between cold and cardiovascular markers also found a negative correlation between hs-CRP and ambient temperature when the ambient temperature was at the lowest temperature below 0 °C^19^. However, till now, there is no definitive explanation for this phenomenon. Besides, the incidence of respiratory and other infections increases in cold weather^20–22^, manifested as an increase in hs-CRP, leading to an increase in systemic and coronary inflammation levels. The preliminary findings have also confirmed the link between influenza infection and ACS. A reduction in recurrent cardiovascular adverse events was observed in ACS patients who received the influenza vaccine^23,24^.

Although several studies have found that blood pressure increases in cold weather^25,26^, in our study, no association was found between cold temperature onset and patients’ admission blood pressure. Prior studies investigating the relationship between cold weather and ACS have also not identified a correlation between blood pressure and weather^14^. Therefore, we hypothesize that the occurrence of MI in cold environments may not be attributed to short-term fluctuations in blood pressure. Besides, studies pointed out that blood pressure is more closely related to indoor temperature or personal-level environmental temperature than outdoor temperature^27,28^. Therefore, the indoor central heating in northern China may affect patients’ true ambient temperature. Furthermore, patients’ blood pressure is not collected immediately at the time of the onset of the disease. This could also be attributed to the absence of statistically significant differences.

Similar to previous studies, the proportion of plaque rupture was higher in cold temperature group^12–14^. In addition, our result found that the minimum fibrous cap thickness was thinner in the cold temperature group. It has always been considered plaque rupture is usually formed by the progression of vulnerable plaques^29^. Rupture of the fibrous cap of the plaque and outflow of the lipid core can lead to rapid thrombus formation leading to luminal obstruction^30^. A thinner fibrous cap and increased levels of vascular inflammation may contribute to plaque instability^31,32^. Furthermore, it is important to note that although the correlation is weak, our analysis revealed a positive correlation between ambient temperature and fibrous cap thickness.

Moreover, we also found that although no differences were seen in the lipid angle and length among the various groups, patients in the cold temperature group were more likely to present with lipid plaques. The main cause of the plaque rupture event is the rupture of the fibrous cap and the outflow of the necrotic core, which leads to thrombosis^33^. Therefore, we speculate that cold may promote the formation of lipid plaque and the reduction of the fibrous cap, leading to a higher incidence of plaque rupture in the cold temperature group^34^. Besides, although one study pointed out that the cholesterol crystals were close to low-temperature^14^, however, this was not found in our study. This variance may be attributed to varying long-term environmental temperatures within distinct geographical locations, implying that the features of cold-related plaque may differ across different environments. Therefore, we should further develop targeted prevention and control strategies based on the actual cold-related cardiovascular hazard mechanisms in each region.

Additionally, our study results demonstrate that despite the better improvement in blood flow of culprit lesions in both groups post-treatment, and a similar observed incidence of microcirculatory dysfunction during the procedure, the 3-year prognosis of patients in cold environments still exhibited a worse outcome. This was mainly manifested in promoting the incidence of MACE events and the recurrence of HF events.

Previous studies have found that patients with plaque rupture are more prone to experience adverse cardiac events compared to those with plaque erosion^35,36^. Additionally, patients with plaque rupture have been observed to exhibit higher levels of coronary inflammation and a greater extent of global coronary artery disease, such as a higher proportion of vulnerable plaques in non-culprit lesions than patients with plaque erosion^31,37,38^. We hypothesize that cold weather may worsen the severity of coronary artery disease in STEMI patients by elevating inflammation levels, promoting unstable coronary artery plaques, and contributing to plaque rupture. Therefore, patients who develop symptoms during cold weather may experience a more severe ischemic condition, leading to the occurrence of long-term MACE events and HF events. It is worth noting that another study suggests that ACS patients with winter onset who underwent an OCT examination showed a higher 2-year cardiac death rate^12^. While our findings also demonstrated an increased incidence of cardiac death events associated with cold temperature exposure, statistical significance was not achieved. This may be due to the limited sample size in the study. Also, it has been shown that insulation can reduce cardiovascular damage from cold exposure^22^. Hence, our results suggest clinicians may consider enhancing management when treating STEMI patients in cold weather, such as anti-inflammatory therapy, to reduce the potential occurrence of adverse cardiovascular events in the long term.

There are limitations to this study. Firstly, it was a retrospective, single-center, observational study. The number of patients included in the study was small and needs to be verified in a larger study population in the future. Second, as all patients had undergone prior OCT imaging for STEMI patients, so there may be a selection bias in the inclusion of the study population. Third, this study only explored the relationship between ambient temperature and coronary atherosclerosis in the disease environment. Environmental pollution-related indicators, such as PM2.5, ozone, and other potential influencing factors, as well as whether to perform complete revascularization and the control of patient risk factors during follow-up, may also impact the lesions and prognosis of patients^39^. Fourth, whether patients included in this study undergo stent treatment is comprehensively decided by the attending physician based on the patient’s actual condition and whether the patient agrees to stent treatment. However, after Cox correction with or without stent treatment as a variable, an adverse effect of cold temperature onset exposure on prognosis was still obtained in our study.

## Methods

### Study design and patient population

This is a single-center retrospective study. In total, 542 STEMI patients who underwent OCT examination within 12 h of admission after emergency coronary angiography and had complete 3-year follow-up information from January 2018 to December 2018, were included in our study. The definition of acute STEMI refers to the Fourth Edition of the Myocardial Infarction Guidelines^40^. 25 patients were excluded for the following reasons: (1) coronary artery dilatation before OCT examination (*n* = 5); (2) poor OCT image quality (*n* = 6); (3) blood not fully flushed before OCT examination (*n* = 3); (4) in-stent restenosis (*n* = 3); (5) no pre-stent OCT images (*n* = 8). Finally, a total of 517 patients were included in this study (Fig. S5). The patients were then divided into two groups according to the median value of the ambient temperature of the day where the disease occurred in all included people, namely the cold temperature group and the warm temperature group. Weather temperature data were collected from the China Meteorological Administration (https://tianqi.911cha.com/). The Ethics Committee of Harbin Medical University’s Second Affiliated Hospital granted approval for this study, and all enrolled patients provided written informed consent.

### Quantitative coronary angiography and OCT image acquisition and analysis

Quantitative coronary angiography (QCA) was analyzed using the Coronary Angiography Analysis System CAAS Workstation 7.4 (Pie Medical Imaging, Netherlands). The main quantitative indicators measured include reference vessel diameter, minimum lumen diameter, diameter stenosis, and lesion length. In this study, thrombolysis in myocardial infarction myocardial perfusion frame count (TMPFC) was utilized to measure myocardial tissue perfusion^41^. TMPFC > 95.5 frames were indicative of abnormal myocardial perfusion in our study^42^.

OCT scan was performed before culprit lesions treatment. All OCT images were analyzed at the core laboratory without knowledge of patient clinical data. OCT imaging was performed using OPTIS (OPTIS Imaging Systems, Abbott, Plymouth, USA) OCT system. The culprit artery in our study was identified by the combination of angiography, electrocardiography, echocardiography, and OCT imaging^40^. Two cardiologists independently conducted OCT analyses using an offline review workstation. Both experts were unaware of the clinical, angiographic, and weather data. The assessment of all lesions was carried out using quantitative and qualitative analyses, as described in previous studies^43–46^. Plaque rupture was defined by the presence of cavity formation with discontinuity of the fibrous cap in the culprit plaque^43^. Definite erosion was defined as the presence of a luminal thrombus overlying an intact fibroatheroma cap. Possible erosion was defined as a luminal surface irregularity at the culprit lesion in the absence of a clear thrombus, or attenuation of an underlying plaque by a thrombus without superficial lipids or calcification immediately proximal or distal to the thrombus site. A lipid plaque was defined as a signal-rich fibrous cap with poorly delineated borders exhibiting attenuation. Fibrous plaque was defined as a homogeneous plaque with high backscattering and low attenuation^44,45^. Calcifications appeared as regions with low scattering and sharp borders. Macrophage under OCT appeared as a strong signal-rich area followed by a heterogeneous backward shadow. Microchannels appeared as tubular structures with diameters of 50–300 µm that were present in at least three consecutive frames. Cholesterol crystals appeared as linear, signal-rich structures. Proximal and distal reference were the sites with the largest lumen area proximal and distal to the lesion. In principle, the nearest frame with luminal border visibility was used to guide the tracing of the lumen area in those frame segments where the luminal border was not visible due to shadowing by a red thrombus^46^. Minimal lumen area was the smallest lumen area within the length of the lesion. Percent area stenosis was calculated as ([mean reference lumen area−minimal lumen area]/mean reference lumen area)×100. For each lipid plaque, lipid length, maximum lipid arc, and minimum fibrous cap thickness were measured. The lipid length was calculated from the number of consecutive frames with dominant lipid plaque constituents. The minimum fibrous cap thickness was measured at the thinnest part of the cap and was reassessed three times, and the average was then calculated. For the quantitative analysis, the cross-sectional OCT images were examined at intervals of 1 mm.

### Clinical follow-up and endpoints

For all patients included in this study, we have specialized clinical investigators to conduct systematic follow-up information surveys by phone or outpatient clinic, respectively, at 1, 3, 6, 9 months, and yearly till 3 years. All involved patients in our study completed 3 years of clinical follow-up. Cardiac death was defined as death due to MI, cardiac perforation or tamponade, arrhythmia or conduction abnormality, surgical complications, or any death with an unexcluded cardiac cause. All events were judged by an independent clinical committee.

### Statistical analysis

Kolmogorov–Smirnov test was used to describe data distribution. Continuous variables were expressed as mean SD or median (interquartile range [IQR]). Categorical data were presented as counts (proportions). Continuous variables were compared using the independent samples Student’s *t*-test or the Mann–Whitney *U* test. Categorical data were compared using the chi-squared or Fisher exact test. Correlation analysis was performed using Spearman’s correlation coefficient test. Cumulative event rates were estimated according to the Kaplan–Meier method. Event curves were compared by the log-rank test. Results have been presented as HR with 95% CI. To detect independent outcome predictors and compute their adjusted HRs, we employed the Cox regression model. In the adjusted model, age, sex, diabetes mellitus, dyslipidemia, hypertension, N-terminal pro-brain natriuretic peptide (NT-proBNP), high-sensitivity C-reactive protein (hs-CRP), and percutaneous coronary intervention with drug-eluting stents were adjusted for. Significant differences in the data were taken into account when the two-tailed *p*-value was <0.05. All statistical analysis was performed with SPSS, version 23.0.

## Supplementary information

Supplementary Table and Figure

## Data Availability

The aggregate data supporting the findings of this study are available from the corresponding author upon reasonable request.
