# Author Correction: Culprit lesion characteristics and prognosis in STEMI with cold onset: an OCT study

**DOI:** 10.1038/s44325-025-00068-4

**Published:** 2025-06-17

**Authors:** Qianhui Sun, Xing Luo, Boling Yi, Chen Zhao, Minghao Liu, Ming Zeng, Haibo Jia, Bo Yu

**Affiliations:** 1 Harbin Medical University, 150081 Harbin, PR China; 2 Department of Cardiology, The 2nd Affiliated Hospital of Harbin Medical University, 150001 Harbin, PR China; 3 National Key Laboratory of Frigid Zone Cardiovascular Diseases, 150001 Harbin, PR China

**Keywords:** Cardiology, Cardiovascular diseases

Correction to: *npj Cardiovascular Health* 10.1038/s44325-024-00019-5, published online 01 October 2024

In this article the Competing Interests statement was incorrect and should have been ‘Author Haibo Jia is a Guest Editor at *npj Cardiovascular Health*. Haibo Jia was not involved in the journal’s review of, or decisions related to, this manuscript.’ The original article has been corrected.

