# Supplementary material for: Culprit lesion characteristics and prognosis in STEMI with cold onset: an OCT study
Source: NPJ Cardiovasc Health. 2024 Oct 1;1:24. doi: 10.1038/s44325-024-00019-5 (PMC12912430; doi:10.1038/s44325-024-00019-5)
Supplement: Supplementary file 1 — Supplementary Table and Figure [file 44325_2024_19_MOESM1_ESM.pdf]

Supplemental Material

Figure S1: Number of patients under different ambient temperatures 1

Figure S2: Plaque characteristics of the cold temperature group and the warm temperature group under OCT observation 2

Figure S3: Correlation analysis between fiber cap thickness and ambient temperature 3

Figure S4: The Kaplan–Meier curve for Rehospitalizations for HF comparing warm temperature group vs cold temperature group 4

Figure S5: Flow chart of patient selection 5

Table S1: OCT characteristics of culprit plaque between extremely cold temperature group and non-extremely cold temperature group 6

Table S2: Events at 3 years stratified by ambient temperature 7

Figure S1: Number of patients under different ambient temperatures

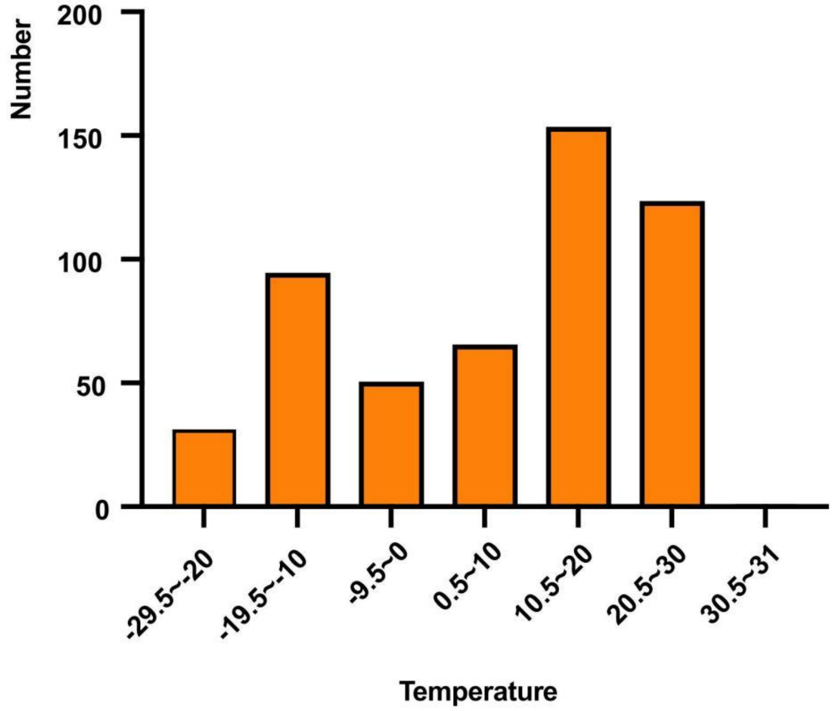

The figure shows the environmental temperature and the number of included patients at the time of disease onset in different patient groups.

Figure S2: Plaque characteristics of the cold temperature group and the warm temperature group under OCT observation

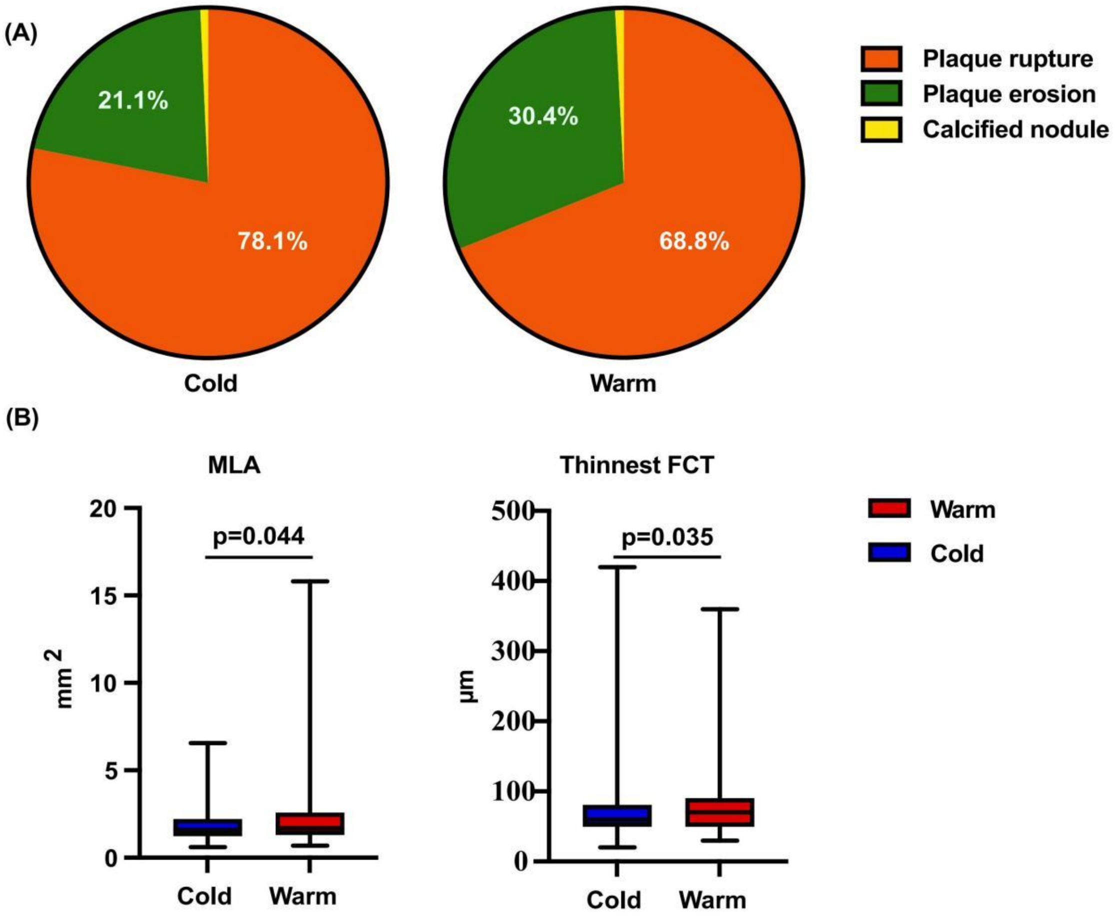

A. The proportion of plaque rupture in the cold temperature group was 78.1%, and the plaque erosion rate was 21.1%. B. In the warm temperature group, 68.8% of plaque ruptures occurred, and 30.4% of plaque erosions occurred. Patients in cold temperature group had smaller minimum lumen area and thinner minimum fibrous cap thickness. MLA= Minimum lumen area； FCT= fibrous cap thickness

Figure S3: Correlation analysis between fiber cap thickness and ambient temperature

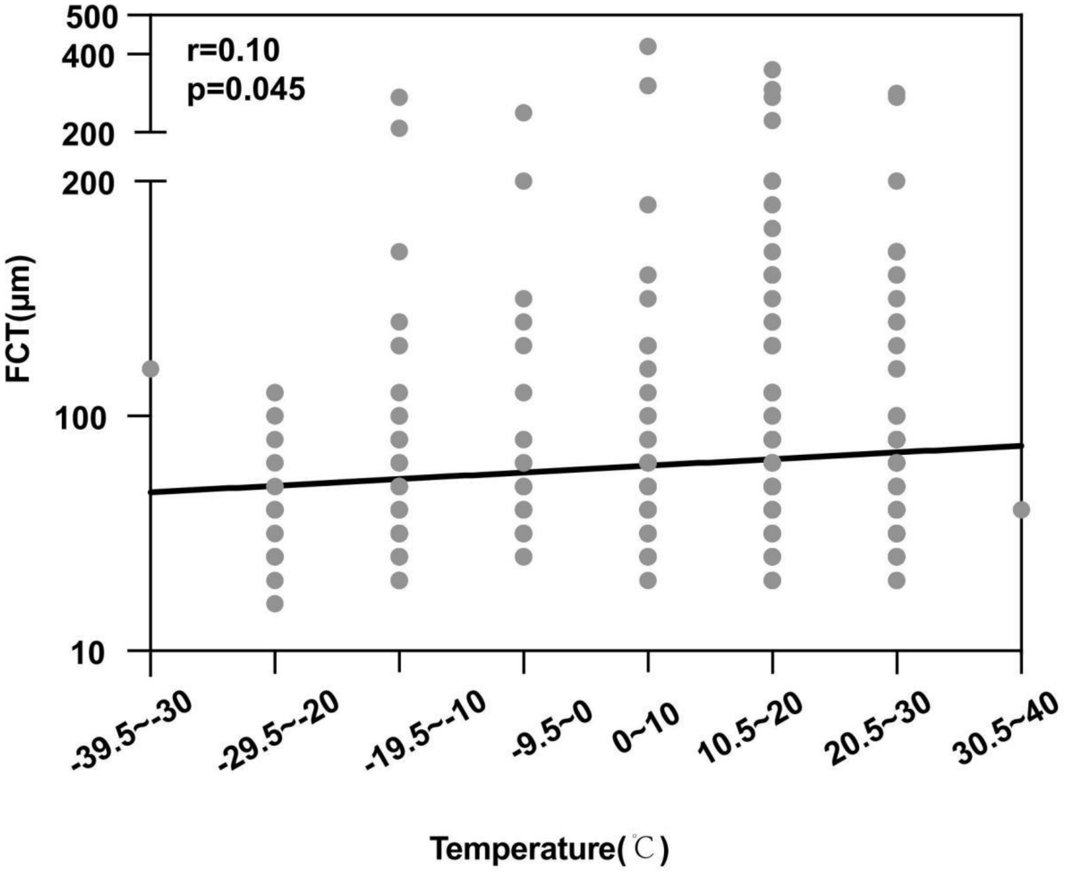

As the ambient temperature decreases, the patient's minimum FCT decreases. The strength of the correlation was measured using Spearman’s correlation coefficient (r). FCT= fibrous cap thickness

Figure S4: The Kaplan–Meier curve for Rehospitalizations for HF comparing warm temperature group vs cold temperature group

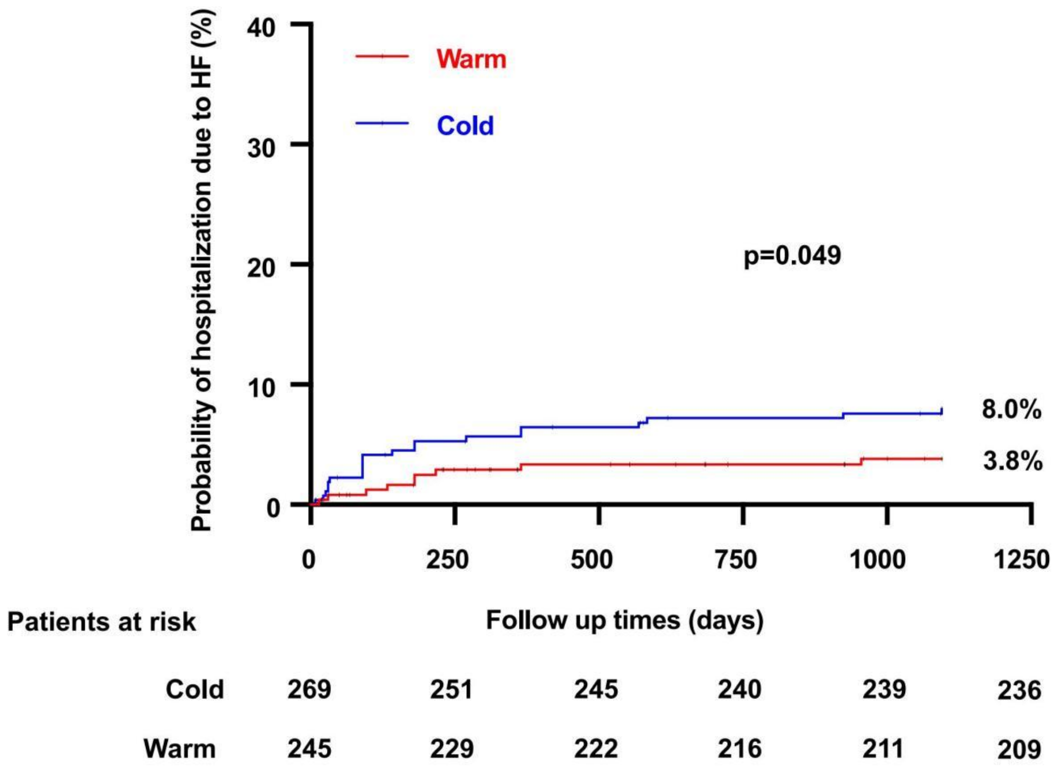

Kaplan-Meier curves for unadjusted incidence of rehospitalizations for HF during the 3-year follow-up period showing the accumulated incidence for rehospitalizations for HF.

HF = heart failure.

Figure S5: Flow chart of patient selection

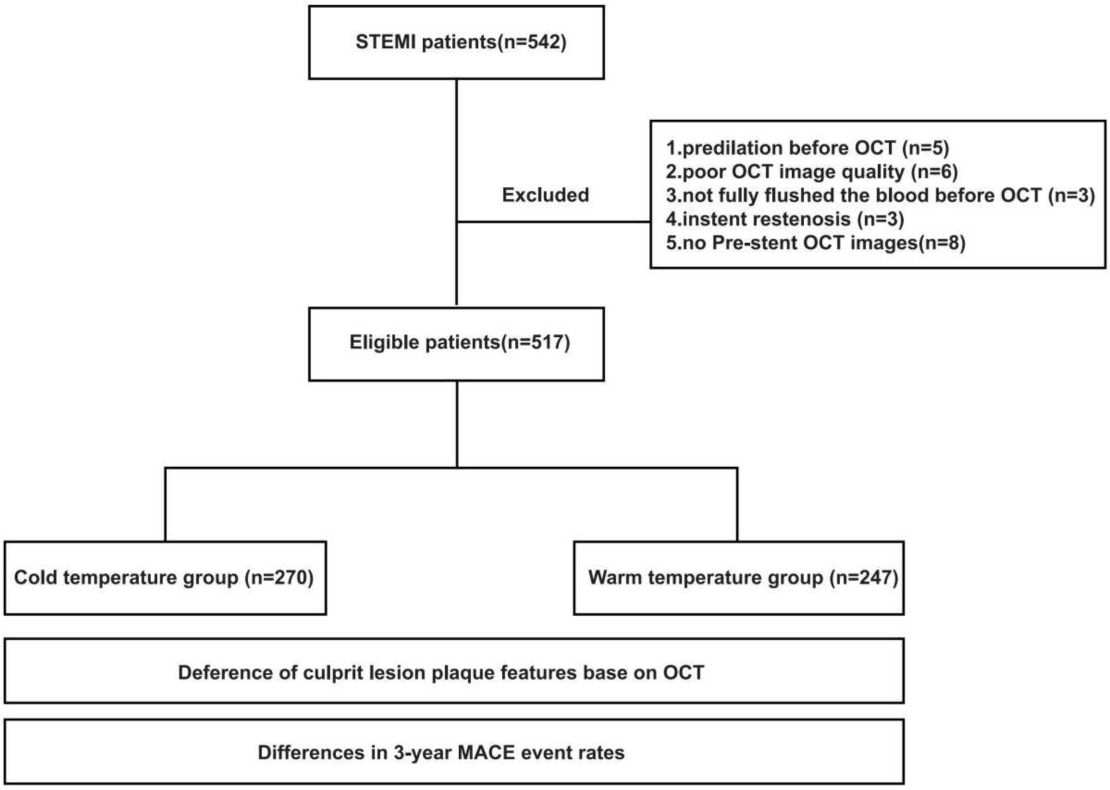

The research flowchart depicts the exclusion criteria and grouping status of this study.

Table S1: OCT characteristics of culprit plaque between extremely cold temperature group and non-extremely cold temperature group

Variables	Extremely cold group (n=58)	Non-extremely cold group (n=459)	p
Plaque rupture, n (%)	49(84.5)	332(72.3)	0.048
Lipid plaque, n (%)	50(86.2)	385(84.1)	0.672
Macrophage, n (%)	53(91.4)	423(92.4)	0.793
Calcification, n (%)	35(60.3)	242(52.8)	0.280
Microvessel, n (%)	11(19.0)	101(22.1)	0.591
Cholesterol crystal, n (%)	16(27.6)	103(22.5)	0.385
MLA, mm2	1.6(1.2-2.4)	1.6(1.3-2.3)	0.804
AS, %	73.7(64.7-81.4)	73.4(63.7-80.8)	0.685
Thinnest FCT, µm	60.0(50.0-80.0)	70.0(50.0-90.0)	0.029
Mean lipid arc, °	163.8(138.7-198.7)	176.7(151.0-210.6)	0.103
Maximal lipid arc, °	254.5(195.3-360)	289.7(229.1-360)	0.368
Lipid length, mm	11.5(8.9-15.2)	10.7(7.5-14.6)	0.733

Values expressed as n (%), mean ± SD, or median (25th-75th percentiles). A p-value < 0.05 was considered statistically significant.

AS = Area stenosis; FCT = fibrous cap thickness; MLA = minimal lumen area; MLD = minimal lumen diameter.

Table S2: Events at 3 years stratified by ambient temperature

	Cold temperature group (n=270)	Warm temperature group (n=247)	p
MACE	42(15.7)	23(9.7)	0.041
Cardiac death	14(5.2)	10(4.2)	0.599
Nonfatal MI/Stroke	11(4.2)	6(2.6)	0.327
Rehospitalizations for HF	21(8.0)	9(3.8)	0.049

MACE = major adverse cardiac events; MI = myocardial infarction; HF = heart failure.
